# Role of second-hand smoke (SHS)-induced proteostasis/autophagy impairment in pediatric lung diseases

**DOI:** 10.1186/s40348-017-0069-7

**Published:** 2017-02-02

**Authors:** Neel Patel, Christopher D. Trumph, Manish Bodas, Neeraj Vij

**Affiliations:** 1 College of Medicine, Central Michigan University, College of Medicine Research Building, 2630 Denison Drive, Room# 120, Room# 120 (Office) and 126-127 (Lab), Mt Pleasant, MI 48859 USA; 2 Department of Pediatric Respiratory Science, The Johns Hopkins University School of Medicine, Baltimore, MD USA

**Keywords:** Cigarette smoke, Second-hand smoke, Proteostasis, Autophagy, Pediatric lung diseases, Bronchopulmonary dysplasia

## Abstract

**Background:**

Exposure to second-hand tobacco smoke (SHS) is one of the prime risk factors for chronic lung disease development. Smoking during pregnancy may lead to birth defects in the newborn that include pulmonary dysfunction, increased susceptibility to opportunistic pathogens, or initiation of childhood respiratory manifestations such as bronchopulmonary dysplasia (BPD). Moreover, exposure to SHS in early childhood can have negative impact on lung health, although the exact mechanisms are unclear. Autophagy is a crucial proteostatic mechanism modulated by cigarette smoke (CS) in adult lungs. Here, we sought to investigate whether SHS exposure impairs autophagy in pediatric lungs.

**Methods:**

Pregnant C57BL/6 mice were exposed to room air or SHS for 14 days. The newborn pups were subsequently exposed to room air or SHS (5 h/day) for 1 or 14 days, and lungs were harvested. Soluble and insoluble protein fractions isolated from pediatric mice lungs were subjected to immunoblotting for ubiquitin (Ub), p62, VCP, HIF-1α, and β-actin.

**Results:**

Our data shows that short-term exposure to SHS (1 or 14 days) leads to proteostasis and autophagy-impairment as evident by significant increase in accumulation of ubiquitinated proteins (Ub), p62 (impaired-autophagy marker) and valosin-containing protein (VCP) in the insoluble protein fractions of pediatric mice lungs. Moreover, increased HIF-1α levels in SHS-exposed mice lungs points towards a novel mechanism for SHS-induced lung disease initiation in the pediatric population. Validating the in vivo studies, we demonstrate that treatment of human bronchial epithelial cells (Beas2b cells) with the proteasome inhibitor (MG-132) induces HIF-1α expression that is controlled by co-treatment with autophagy-inducing drug, cysteamine.

**Conclusions:**

SHS-exposure induced proteostasis/autophagy impairment can mediate the initiation of chronic lung disease in pediatric subjects. Hence, our data warrants the evaluation of proteostasis/autophagy-inducing drugs, such as cysteamine, as a potential therapeutic intervention strategy for SHS-induced pediatric lung diseases.

## Background

Second-hand cigarette smoke (SHS) is a major environmental risk factor affecting the pediatric population. Over five decades ago, it was discovered that cigarette smoking during pregnancy impacts fetal development leading to premature birth and other birth defects [1, 2]. However, a recent study from the UK showed that mothers exposed to SHS also demonstrate an increased risk of early delivery and reduced birth weight of infants [3]. Gestational SHS exposure is known to induce irreversible hypoalveolarization and decreased angiogenesis, and lung secretory function, leading to bronchopulmonary dysplasia (BPD). Moreover, children exposed to SHS during their early phases of development are more susceptible to infections that may trigger inflammation and promote BPD pathogenesis. Although, underlying mechanisms and outcomes of BPD are not thoroughly investigated in the pediatric population.

Cigarette smoke (CS) is known to modulate the ubiquitin-proteasome pathway that is crucial in maintaining proteostasis [4, 5]. The initial CS-mediated activation of the ubiquitin-proteasome pathway triggers the unfolded protein response so that misfolded proteins can be either recycled, folded properly, or degraded. However, chronic-CS exposure induces the accumulation of insoluble ubiquitinated proteins, at rates faster than the proteasome is able to degrade them. Moreover, sequestosome-1/p62, a marker for autophagy impairment, is elevated in chronic smokers [6, 7] and its accumulation in the insoluble fraction indicates its localization to aggresome-bodies [6, 7]. Valosin-containing protein (VCP) can facilitate degradation of misfolded proteins but its accumulation in aggresome-bodies on CS exposure [6] impairs both autophagy and proteostasis mediated clearance of misfolded proteins.

CS exposure is known to induce hypoxia inducible factor-1 (HIF-1α) expression in murine and rabbit lungs. Briefly, HIF-1α is a transcription factor that mediates pulmonary response to hypoxia as its expression is closely regulated by oxygen concentration, which is largely responsible for the HIF-1α transcriptional activity [8, 9]. Hence, CS-induced HIF-1α expression contributes to the pathogenesis of chronic obstructive pulmonary disease (COPD) [8] and therefore can also play a role in the development of BPD. Interestingly, overexpression of HIF-1α in the embryonic pulmonary epithelium leads to impaired branching morphogenesis and decreased epithelial cell proliferation, which negatively impacts lung maturation [10]. This impairment of lung maturation when coupled with mechanical ventilation post-premature birth is thought to promote the susceptibility of such a patient population to develop BPD [11]. Thus, we designed this preliminary study to identify the effects of CS/SHS on HIF-1α expression, proteostasis/autophagy, and its potential impact on development of pediatric lung disease(s) such as BPD.

## Correspondence/Findings

Briefly, we used a pediatric and neonatal murine model to identify the effects of CS/SHS exposure on proteostasis/autophagy and HIF-1α expression as a potential mechanism for development of BPD-like pulmonary dysfunction in the pediatric population.

## Methods

### Mice SHS exposure

All animal experiments were performed following our Institutional (JHU and CMU) Animal Care & Use Committee (IACUC) protocols. Pregnant C57BL/6 mice were exposed to room air or SHS for 5 h/day for 14 days as we have previously described in detail [12]. After delivery, the pediatric mice were similarly exposed to either room air or SHS for one day, and likewise, another group was exposed to either conditions for 14 days. After the respective exposures, the mice were sacrificed and the pediatric lungs were harvested for further analysis by immunoblotting.

### Western blot analysis

After room air and SHS exposure, the lung tissues were harvested and lysates were prepared using RIPA buffer (25 mM Tris-HCl [pH 7.6], 150 mM NaCl, 1% NP-40, 1% sodium deoxycholate, 0.1% SDS) containing 1× protease inhibitor cocktail (Pierce). Lung tissues were homogenized and sonicated on ice for 3–5 min and with 10–15 s pulses, respectively. The Bradford Protein Assay Kit was used to quantify the amount of protein in the total protein lysates. Soluble and insoluble protein fractions were separated by centrifugation at 13,000 rpm for 15 min at 4 °C. Soluble (50 μg) and/or insoluble protein fractions (pellet, isolated from equal amount of protein 500 μg, for each sample) were separated on 10% SDS-PAGE and transferred to 0.45 μm nitrocellulose membranes (Bio-Rad) for immunoblotting. The membranes were blocked with 1% nonfat dry milk at room temperature for 1 h on a rotary shaker, followed by overnight incubation at 4 °C with mouse monoclonal ubiquitin (1:1000; Santa Cruz), rabbit polyclonal p62 (1:500; Santa Cruz), rabbit polyclonal VCP (1:500; Santa Cruz), rabbit polyclonal HIF-1α (1:500; Santa Cruz), and mouse monoclonal β-actin (1:5000; Sigma) as primary antibodies. The membranes were washed (3×) with a PBS-Tween buffer (0.5% tween-20 in 1× PBS) and incubated with 1:6000 goat horseradish peroxidase-conjugated anti-mouse antibody (Novus Biologicals) or 1:6000 goat horseradish peroxidase-conjugated anti-rabbit antibody (Novus Biologicals) for 1 hr at room temperature, followed by 3× washes with PBS-tween buffer as described above. The Clarity™ Western ECL substrate (Bio-Rad) was used for chemiluminescence detection of immunoblots using a LI-COR C-DiGit™ Blot Scanner. Images captured with Image Studio Lite 5.0, and ImageJ 1.49o (NIH) software were used to quantify changes in protein expression by densitometry analysis and using β-actin (soluble fraction) as the loading control.

### In vitro experiments

The human bronchial epithelial cells (Beas2b cells) were cultured using standard cell culture conditions as recently described [13]. The cells were plated in six well plates and treated with MG-132 (5 μM) and/or cysteamine (250 μM) for 12 h. Cell lysates were prepared using the RIPA buffer, and equal amount of total protein was separated on a 10% SDS-PAGE and transferred to 0.45 μm nitrocellulose membranes (Bio-Rad) for immunoblotting as described above. Briefly, the membranes were immunoblotted for HIF-1α (rabbit polyclonal; 1:500; Santa Cruz) or β-actin (mouse monoclonal;1:5000; Sigma) primary antibodies followed 1:6000 goat horseradish peroxidase-conjugated anti-mouse antibody (Novus Biologicals) or 1:6000 goat horseradish peroxidase-conjugated anti-rabbit antibody (Novus Biologicals).

## Results

### Second-hand cigarette smoke (SHS) exposure causes proteostasis/autophagy impairment in neonatal mice lungs

We have recently shown that tobacco-smoke, e-cigarette vapor (eCV), and/or inhaled-nicotine exposure induces proteostasis/autophagy impairment as a central mechanism for pathogenesis of COPD-emphysema [4, 6, 7, 14]. Hence, we were interested in evaluating this as a potential mechanism of SH tobacco-smoke exposure on the pediatric population.

**Fig. 1:**
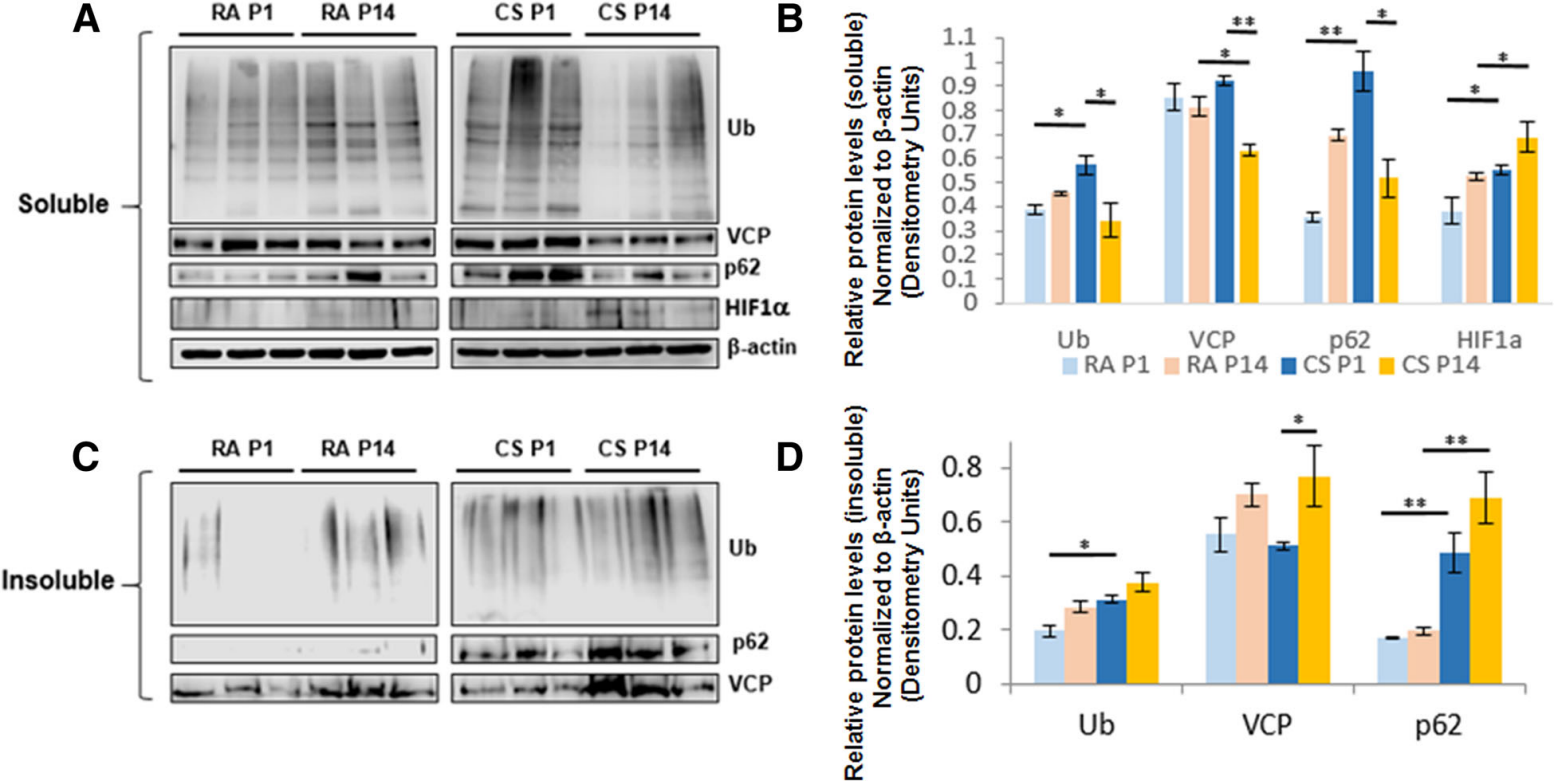
SHS exposure induces autophagy-impairment in pediatric and neonate murine lungs mice lungs. **a**, **c** Western blots showing the accumulation of ubiquitinated proteins (ubiquitin, Ub), p62 (sequestosome-1), VCP (valosin containing protein), and HIF-1α (hypoxia inducible factor) in the soluble and/or insoluble lung protein-fractions isolated from SHS (CS) or room air (RA)—exposed neonatal mice sacrificed at day 1 (P1) or 14 (P14), post-delivery. The mothers of these pups were also exposed to SHS for the last 14 days of the pregnancy. **b** *Bar graph* of relative optical density of soluble Ub, VCP, p62, and HIF-1α normalized to β−actin. **d** *Bar graph* of relative optical density of insoluble Ub, VCP, and p62 is also normalized to soluble β-actin. (*p < 0.05; **p < 0.01)

Here, we report that SHS exposure of pregnant mice (14 days) followed by exposure to the pups (either 1 or 14 days), leads to significant (*p* < 0.05) accumulation of ubiquitinated proteins in the soluble and insoluble protein fractions of pediatric mice lungs, as compared to room air (RA) exposed mice, indicating early initiation of autophagy-impairment (Fig. 1a–d, *p* < 0.05). The data further indicates that 14 days of SHS exposure to neonatal mice induces higher accumulation of ubiquitinated proteins in the insoluble protein fraction as compared to 1-day exposure (Fig. 1c, d), implying increase in proteostasis and/or autophagy-impairment in 14-day-exposed mice lungs.

We have shown earlier that autophagy-impairment leads to accumulation of VCP and p62 in the insoluble protein fractions (aggresome bodies) [6, 7]. In accord with previous findings, here, we report that both day 1 and 14-SHS-exposure groups of mice show significantly higher p62 protein levels (insoluble), and a moderate increase in VCP in the insoluble protein fraction as compared to room air control (Fig. 1c, d), suggesting autophagy-impairment.

**Fig. 2:**
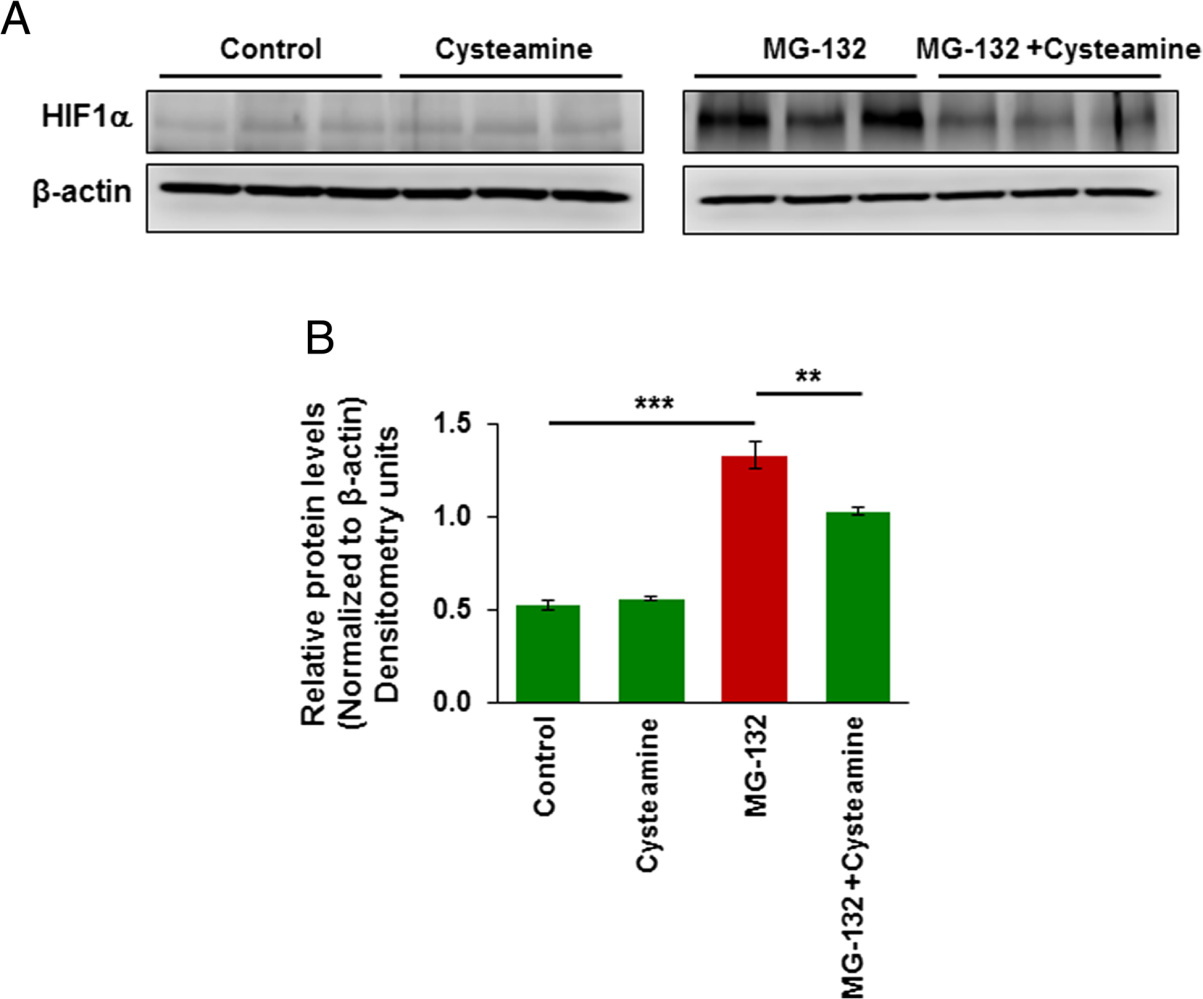
Proteasome inhibition induced HIF-1α level is controlled by autophagy inducing drug, cysteamine. **a**, **b** Western blots showing the changes in HIF-1α expression in total protein lysates isolated from Beas2b cells treated with MG-132 (5 μM) and/or cysteamine (250 μM) for 12 h. The data (mean ± SEM, *n* = 3) indicates that MG-132 treatment significantly elevates HIF-1α levels that were reduced by co-treatment with autophagy-inducing drug, cysteamine. (**p < 0.01; ***p < 0.001)

Additionally, a significant increase in HIF-1α levels is observed in 1-day-SHS exposed mice compared to 1-day room air control (*p* < 0.05). Moreover, after 14 days of SHS exposure, there is even more significant (*p* < 0.05) change in HIF-1α as compared to 14-day room air controls. Data suggests that HIF-1α may be elevated to control the acute response to SHS exposure. Previous studies have shown that HIF-1α is degraded via the ubiquitin-proteasome system [15]. We also observed a significant increase in HIF1-α levels in Beas2b cells upon proteasome inhibition using MG-132, which was restored by treatment with an autophagy-inducing drug, cysteamine (Fig. 2a, b, *p* < 0.05), indicating that HIF-1α levels could be modulated by proteostasis/autophagy impairment. Overall, these findings suggest that even acute exposure to SHS during pregnancy (14 days; day 1 group) may lead to the impairment of crucial proteostasis/autophagy mechanisms potentially rendering the neonate/pediatric population more susceptible to pulmonary diseases such as BPD.

## Discussion

**Fig. 3:**
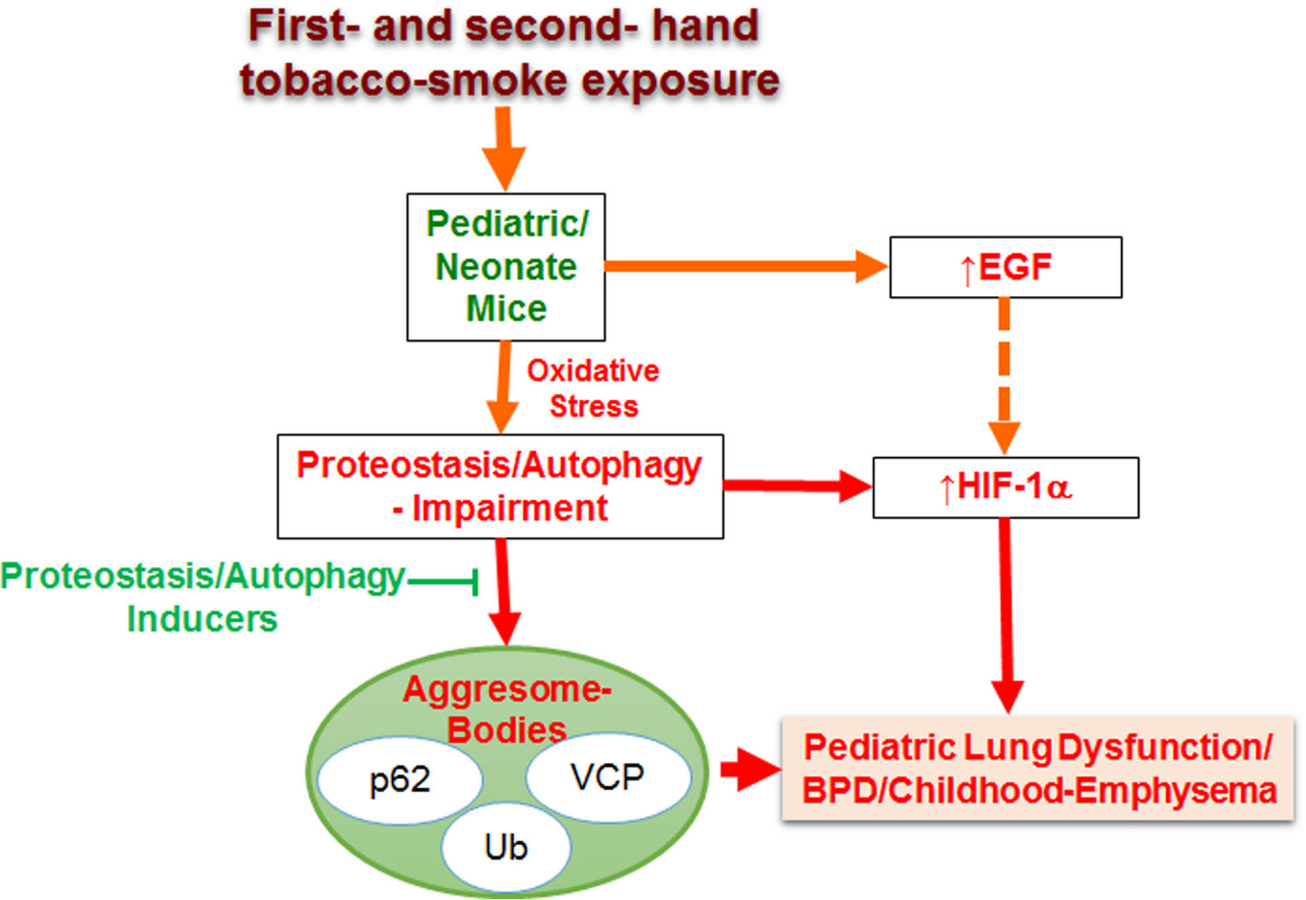
Schema showing novel mechanism for SHS-induced pediatric lung dysfunction. Exposure to SHS during early lung development (neonates/pediatric population) may potentiate initiation of chronic lung disease via oxidative stress mediated proteostasis/autophagy impairment and/or induction of HIF-1α levels. Therapeutic intervention using proteostasis/autophagy inducing drugs may mitigate the SHS exposure-mediated pathogenic changes initiating pediatric lung diseases such as bronchopulmonary dysplasia (BPD) or adult COPD-emphysema-like childhood condition

We report here that similar to the deleterious effects of SHS exposure on adult pulmonary function, acute SHS exposure of pediatric mice born after an in utero SHS exposure (14 days during pregnancy) can potentially lead to initiation of lung disease via autophagy-impairment mediated aggresome formation that is known to promote the pathogenesis of chronic obstructive lung disease (Fig. 3) [6, 7].

Our preliminary results show that proteostasis/autophagy impairment caused by SHS occurs as early as day one of birth after SHS exposure during pregnancy. This defect may have severe consequences on the development of the lungs and can lead to pediatric lung conditions, such as BPD. We have previously shown that an early age-related (pediatric vs adult) decline in proteostasis augments NFκB-mediated inflammation; *Pa*-LPS-induced acute lung injury (ALI) and sepsis [16]. SHS exposure in young children can similarly initiate an early decline in proteostasis in developing lungs, thus increasing the susceptibility to pulmonary exacerbations. SHS is also known to induce acquired-cystic fibrosis transmembrane conductance regulator (CFTR) dysfunction via proteostasis/autophagy impairment [17] but the underlying mechanisms and outcomes are not thoroughly investigated in the pediatric population and represent a future area of study. Since cystic fibrosis (CF) is a known pediatric genetic disease, there is emphasis on the need to evaluate SHS-induced acquired-CF in preterm/pediatric patients. A few recent studies demonstrate that modulating proteostasis could help in the rescue of the misfolded ΔF508-CFTR protein [18] thus highlighting the therapeutic potential of proteostasis augmentation in cystic fibrosis lung disease [19, 20]. Furthermore, SHS impairs bacterial phagocytosis in macrophages by diminishing CFTR expression in the lipid rafts, resulting in increased susceptibility to infections [17]. Thus, SHS-induced prolonged infection and an exaggerated inflammatory response may facilitate the initiation of chronic lung diseases in pediatric subjects.

Moreover, deficiency of membrane CFTR also causes autophagy-impairment via induction of reactive oxygen species (ROS) [12], leading to peri-nuclear accumulation of damaged/misfolded proteins in aggresome-bodies, as seen by the accumulation of ubiquitinated proteins, p62 and VCP in the insoluble fraction, which may mediate COPD-like symptoms or BPD in pediatric subjects. During fetal development, baseline autophagy is associated with maintenance of healthy pregnancy, while autophagy-impairment can promote preterm delivery [2]. This may explain the higher susceptibility of preterm infants to respiratory infections, as a robust autophagy response is essential for clearance of common lung pathogens like *Pseudomonas aeruginosa* (*Pa*). Hence, restoring autophagy-impairment may be a lucrative therapeutic strategy to alleviate COPD-like symptoms and prevent BPD in SHS-exposed infants.

The underlying mechanism(s) of in utero SHS-induced alterations in airway physiology is yet to be studied and may involve proteostasis-mediated modulation of surfactant proteins, NFκB and growth factors. Specifically, SHS is known to induce NFκB levels by regulating proteasomal degradation of its endogenous inhibitor, IκB [21], which is anticipated as the potential mechanism for NFκB induction in BPD, similar to its role in other diseases states such as COPD, cystic fibrosis (CF), etc. In BPD, NFκB is also known to inhibit expression of fibroblast growth factor (FGF-10) that is essential for branching and morphogenesis of the conducting airways of the lungs [22]. NFκB induction, via SHS exposure, can reduce FGF-10 expression in preterm infants promoting initiation of BPD [23].

Altered expression of other common growth factors such as vascular endothelial growth factor (VEGF) and epidermal growth factor (EGF) are also associated with BPD pathogenesis and are regulated by autophagy mechanisms [22]. VEGF is a known potent mitogen for the induction of vascularity, and its expression is downregulated in BPD that may decrease nitric oxide (NO) levels, which is known to promote angiogenesis during lung development [22, 24]. Interestingly, SHS is known to decrease VEGF and NO production, while autophagy induction, specifically, autophagy related protein-7 (Atg7), enhances NO production by the JAK/STAT pathway, which may promote normal lung development [25]. Another growth factor, EGF, essential for lung development is overexpressed in BPD, which then adversely impacts alveolar septa-formation [23]. Moreover, EGF has been shown to inhibit autophagy that can impact lung development by changing levels of NO. In fact, HIF-1α expression is acutely modulated by the epidermal growth factor (EGF) receptor-mediated cell signaling [26]. The early elevation of HIF-1α in our model may be explained by the SHS-induced EGF. EGF is also essential for lung development and is overexpressed in BPD, which then adversely impacts alveolar septa-formation [23], further supporting our hypothesis regarding the mechanism for SHS-induced BPD (Fig. 3).

There is a paucity of information regarding the mechanisms by which SHS-mediated proteostasis impairment induces variety of pediatric lung diseases. Our data shows the extent of SHS exposure-induced proteostasis/autophagy impairment that can occur post-delivery at a very young age. Hence, further research is warranted to validate the potential benefits of proteostasis/autophagy regulators in mitigating SHS-induced inflammatory apoptotic stress responses, pulmonary exacerbations, and/or chronic lung diseases in infants and preterm babies.

### Implications

Chronic SHS exposure is known to impact normal lung development via alterations in growth factors, and frequent occurrence of acute respiratory problems, which may lead to initiation of chronic lung disease. Our data shows that even acute exposure to SHS in utero causes proteostasis/autophagy impairment in the pediatric/neonate murine lungs as a plausible mechanism to trigger progression of chronic lung disease(s). Thus, restoring proteostasis/autophagy could be of potential therapeutic advantage for SHS-mediated pediatric lung disease(s) in pediatric/neonate subjects.

## Abbreviations

ALI: Acute lung injury
Atg7: Autophagy related protein-7
BPD: Bronchopulmonary dysplasia
CF: Cystic fibrosis
CFTR: Cystic fibrosis transmembrane conductance regulator
COPD: Chronic obstructive pulmonary disease
CS: Cigarette smoke
eCV: E-cigarette vapor
EGF: Epidermal growth factor
FGF-10: Fibroblast growth factor-10
HIF-1α: Hypoxia inducible factor-1 alpha
LPS: Lipopolysaccharide
NFκB: Nuclear factor kappa-light-chain-enhancer of activated B cells
NO: Nitric oxide
p62: Sequestosome-1
*Pa*: *Pseudomonas aeruginosa*
RA: Room air
ROS: Reactive oxygen species
SHS: Second-hand smoke
Ub: Ubiquitin
VCP: Valosin containing protein
VEGF: Vascular endothelial growth factor
β-actin: Beta actin
